# Editorial: Molecular Mechanisms of Metalloid Transport, Toxicity and Tolerance

**DOI:** 10.3389/fcell.2018.00099

**Published:** 2018-08-28

**Authors:** Gerd Patrick Bienert, Markus J. Tamás

**Affiliations:** ^1^Metalloid Transport, Department of Physiology and Cell Biology, Leibniz Institute of Plant Genetics and Crop Plant Research (IPK), Gatersleben, Germany; ^2^Department of Chemistry and Molecular Biology, University of Gothenburg, Göteborg, Sweden

**Keywords:** metalloid, transport, toxicity, arsenic, boron, antimony, tellurium, silicon

Metalloids are elements that possess physical and chemical properties that are intermediate between metals and nonmetals. Six elements are commonly designated as metalloids; arsenic (As), antimony (Sb), boron (B), germanium (Ge), silicon (Si), and tellurium (Te). Certain other elements, like astatine (At) and selenium (Se), are sometimes added to the list. Some metalloids have been in use since ancient times, for instance for medicinal purposes. Today, metalloids are mainly used by the industry as constituents of semiconductor devices, ceramics, solar batteries, certain polymers, construction material as well as in medicine and agriculture. Beside geological sources, mining activities and industrial applications may result in high local concentrations of metalloids, rising concerns for human and environmental health. Metalloids affect living organisms in multiple ways. B, Si, and Se have either essential or important functions in most organisms including humans. B and Se may also become toxic when present at high concentrations. In contrast, As, Sb, Te, and Ge are highly toxic and exposure can cause various physiological dysfunctions, growth defects, and human diseases. Despite their toxicity, As and Sb have long histories of usage as chemotherapeutic agents and are important constituents of currently used pharmacological drugs. The impact of metalloids on the environment, agriculture, and human health underscores the importance of elucidating the transport pathways by which metalloids enter, exit, or redistribute within cells and organisms as well as toxicity and tolerance mechanisms. This Research Topic comprises six Original Research Articles and one Review covering various aspects of metalloid biology ranging from the pathophysiology of metalloids and the use of metalloids in medical therapy to resistance mechanisms in microbes and plants.

Contamination of soils and water by As is widespread and it is estimated that more than 100 million people worldwide are exposed to concentrations above the WHO guideline (Clemens and Ma, 2016). Exposure to As can cause various forms of cancer but the underlying mechanisms have not been completely clarified. To this end, Broberg and colleagues (Ameer et al.) assessed As metabolism in two Argentinean populations exposed to As through drinking water. The authors found that As was associated with two cancer risk markers; increased copy number of mitochondrial DNA and altered telomere length, particularly in individuals with a less-efficient As metabolism. Thus, individuals with lower As biotransformation capacity may have increased susceptible to As-induced toxicity and carcinogenicity.

Polluted soils may be cleaned through the uptake of toxic elements by plants. To implement this phytoremediation concept, several molecular aspects of metalloid biology need to be understood. Sandalio and colleagues (Souri et al.) provided an overview of As uptake, translocation, chelation, and detoxification mechanisms in As hyper-accumulator plants. This knowledge represents the basis for future phytoremediation approaches.

In contrast to plants, microbial communities can thrive in environments containing high concentrations of As (Zhu et al., 2014; Yang and Rosen, 2016). Arsène-Ploetze and colleagues (Hovasse et al.). detected the presence of several species of the bacterium *Thiomonas* in an As-rich acid mine drainage system. The authors also detected the presence of *Thiomonas* arsenite oxidases; major enzymes involved in the oxidation of As(III) to As(V) for energy generation and/or detoxification purposes.

The increasing use of Sb in modern technology applications and for medical treatments calls for a better understanding of the biology of this metalloid (Tamás, 2016). To this end, Wang and colleagues (Liu et al.) studied Sb oxidation and its role in microbial resistance. The authors showed that the IscR transcription factor contributes to Sb oxidation and resistance in the bacterium *Comamonas testosteroni S44*, possibly by modulating oxidative stress responses and Fe-S cluster biogenesis.

Treatment of severe diseases such as Leishmaniasis, sleeping sickness or Schistosomiasis caused respectively by the protozoan parasites *Leishmania, Trypanosoma*, or parasitic worms, involves pentavalent Sb-containing drugs. Drug resistance is increasing but the mechanisms involved are not fully understood (Ponte-Sucre et al., 2017). To this end, Frézard and colleagues ((Dos Reis et al.) characterized transport routes of Sb uptake and release in sensitive and resistant *Leishmania* parasites. The authors suggest that both influx and efflux pathways contribute to diminished cellular levels of Sb and to drug resistance.

B is an essential element for seed plants and probably of high importance for human and animal health and development (Khaliq et al., 2018). Both B deprivation and B excess is detrimental for many physiological processes; however the underlying mechanisms are mostly unknown. In contrast, the intercellular transport of B in plants is well-studied. Two transporter protein families, namely the BOR transporter family and the Nodulin26-like Intrinsic Protein family fine-tune B uptake and efflux both during deficient and toxic conditions and adjust B levels and distribution according to the nutritional demand of the plant (Yoshinari and Takano, 2017). B deficiency as well as B toxicity challenge crop production each year and cause yield losses worldwide. Takano and colleagues (Wakuta et al.) demonstrated that BOR1 proteins represent promising molecular targets to engineer plants with tolerance to excess B by excluding B from the cytosol of shoot cells. Optimizing transport and utilization traits in crop plants regulating the B nutritional status is currently pursued by various plant nutrition and breeding groups.

Certain chemical Te species, such as the tellurite oxyanion ${\text{TeO}}_{2}^{-3}$, are highly toxic for most organisms (Lemire et al., 2013). The molecular mechanisms of tellurite toxicity and resistance remain to be fully understood. Turner and colleagues (Vrionis et al.) investigated the effect of selenite on tellurite toxicity in the bacterium *Escherichia coli*. The authors found that co-exposure to selenite strongly increased bacterial resistance to tellurite. Potential mechanisms of this protective effect of selenite are discussed.

In summary, the papers published in this Research Topic have contributed to our understanding of metalloid transport, toxicity, and tolerance in various organisms. Such knowledge is required to successfully face future societal challenges related to the diverse and increasing spectrum of industrial, technological, and medical applications of metalloids.

## Author contributions

GB and MT made substantial, direct and intellectual contributions to the work, wrote it together and approved it for publication.

### Conflict of interest statement

The authors declare that the research was conducted in the absence of any commercial or financial relationships that could be construed as a potential conflict of interest.
